# Editorial: Ecosystems-centered health and care innovation

**DOI:** 10.3389/fmed.2025.1567993

**Published:** 2025-02-21

**Authors:** Adamantios Koumpis, Panagiotis D. Bamidis, Elisio Costa, Evdokimos Konstantinidis

**Affiliations:** ^1^University Hospital of Cologne, Cologne, Germany; ^2^School of Medicine, Aristotle University of Thessaloniki, Thessaloniki, Greece; ^3^Faculty of Pharmacy, University of Porto, Porto, Portugal

**Keywords:** innovation, innovation ecosystems, collaboration, digital transformation in health, European Health Data Space (EDHS)

## Introduction

The rapid evolution of healthcare technologies has reshaped the way services are delivered, regulated, and perceived by stakeholders. From semantic interoperability in electronic health records to cloud-based regulatory platforms and precision medicine, these innovations aim to optimize outcomes for patients and providers alike. However, they also present challenges—ranging from ethical dilemmas to implementation barriers—that must be addressed to realize their full potential.

This editorial integrates findings from ten influential studies that explore various facets of healthcare innovation, offering a comprehensive view of the advancements and challenges in the field. These studies reflect the dynamic interplay of technology, ethics, policy, and patient-centric design in creating resilient and sustainable healthcare systems.

## Semantic interoperability and data integration

The work of Bossenko et al. emphasizes the foundational role of semantic interoperability in enabling efficient healthcare delivery and secondary data use. Tools developed for transforming health data formats, such as the transition from CDA to FHIR in Estonia, exemplify the potential of reusable, domain-expert-friendly solutions to address interoperability challenges. Similarly, Bregonzio et al.'s work on FAIRification and data fusion within distributed analytics platforms highlights the importance of creating scalable, reusable, and interoperable data infrastructures. Together, these contributions underline the significance of harmonized data frameworks in driving global healthcare innovation.

## Ethical considerations in precision medicine

Ahmed et al. systematic review examines ethical concerns surrounding precision medicine, emphasizing the patient perspective. Key themes include privacy, economic impacts, informed consent, and the risk of discrimination. Addressing these ethical issues requires proactive patient education, research, and policy reforms to build trust and mitigate risks. These findings complement the recommendations by Kurihara et al., who advocates for a data-driven, participant-centered approach in research ethics, reinforcing the value of dynamic consent, and open science practices in fostering inclusivity and transparency.

## Regulatory and ecosystemic approaches

Khalil et al.'s exploration of cloud-based regulatory platforms underscores their potential to revolutionize drug development by expediting the review process and enhancing global accessibility. However, realizing this potential requires concerted policy efforts and technological readiness. Sturmberg et al.'s critique of reductionist research methodologies highlights the need for ecosystemic approaches to clinical decision-making, emphasizing the integration of complex, multi-level health determinants.

## Innovative solutions for aging and dementia care

Figueiredo et al.'s work on Living Labs showcases the value of end-user engagement in designing dementia care solutions. By addressing challenges such as sustainability and scalability, her proposed guidelines aim to maximize the impact of these collaborative innovation methods. Carriazo et al.'s analysis of the Andalusian digital health strategies further illustrates the power of Quadruple Helix collaboration in driving health improvements through ecosystemic approaches.

## The European Health Data Space and global health implications

Laleci Erturkmen et al. and Katehakis et al. focus on the European Health Data Space (EHDS) initiative and the smartHEALTH European Digital Innovation Hub, respectively. Their work underscores the importance of harmonized data standards and interdisciplinary collaboration in advancing precision medicine and AI-driven healthcare services. These initiatives highlight the potential of unified data ecosystems to enhance global clinical research and improve patient outcomes.

## Conclusion

The synthesis of these studies reveals a shared vision for the future of healthcare: one that leverages technology and innovation to improve patient outcomes, empower stakeholders, and address ethical and systemic challenges. Achieving this vision requires a collaborative, interdisciplinary approach, integrating the principles of semantic interoperability, precision medicine, and ecosystemic innovation. By prioritizing ethical considerations and fostering global partnerships, the healthcare community can ensure a sustainable and equitable future for all.
